# The Impact of Non-Dysentery Shigella Infection on the Growth and Health of Children over Time (INSIGHT)—A Prospective Case–Control Study Protocol

**DOI:** 10.3390/microorganisms12081677

**Published:** 2024-08-15

**Authors:** Subhra Chakraborty, Sampa Dash, Nowrin Akbar Antara, Bharati Rani Roy, Shamim Al Mamun, Mohammad Ali, Farina Naz, Fatema-Tuz Johura, Jade Lewis, Farzana Afroze, ABM Ali Hasan, David A. Sack, Malathi Ram, Fahmida Tofail, Tahmeed Ahmed, A. S. G. Faruque

**Affiliations:** 1 Department of International Health, Bloomberg School of Public Health, Johns Hopkins University, Baltimore, MD 21205, USA; 2 icddr,b, Dhaka 1212, Bangladesh; sampa.somc@gmail.com (S.D.); nowi.khan@gmail.com (N.A.A.); farina.naz@icddrb.org (F.N.);; 3 Kumudini Women’s Medical College Hospital, Mirzapur, Tangail 1940, Bangladesh

**Keywords:** *Shigella*, diarrhea, treatment, antibiotics, intestinal inflammation, systemic inflammation, malnutrition, cognitive development, RLDT, diagnostics

## Abstract

(1) *Shigella* spp. (Shigella) is known for causing dysentery with blood in stool, but most children infected with Shigella have non-dysentery Shigella-associated diarrhea (NDSD). The World Health Organization recommends the use of antibiotics when diarrhea is bloody, leaving most NDSD cases untreated. The absence of dysentery may not indicate a low risk of death and severe morbidity. Rapid diagnosis and treatment of shigellosis in vulnerable, young children may be lifesaving. INSIGHT aims to determine the potential benefit of identifying NDSD cases (n = 296) and their outcomes compared to cases of *Shigella dysentery* [DS (n = 148)] and non-Shigella watery diarrhea [WD (n = 148)]. (2) Children seeking care at hospitals in Bangladesh will be enrolled and followed for 1 year (NDSD and DS) or 90 days (WD). We will determine the impact of NDSD on morbidity, mortality, gut health, nutritional status, and cognitive development compared to DS and WD in children and whether the simple “Rapid LAMP-based Diagnostic Test (RLDT)” can accelerate the detection and treatment of shigellosis in the clinical settings of rural Bangladesh. (3) INSIGHT will determine the impact of NDSD in children and determine if the treatment guidelines of shigellosis need to be revisited to improve clinical outcomes and the development of these children.

## 1. Introduction

*Shigella* spp. (Shigella) is a bacterial pathogen that is frequently associated with diarrheal disease and is a significant cause of mortality and morbidity worldwide. Although the mortality associated with shigellosis has decreased in current years to ~70,000 [1,2] in children, there are at least 80 million cases of shigellosis each year worldwide, with the estimated disability-adjusted life years (DALYs) at 7 million and year loss due to disability (YLD) at 744,000 [1,2,3,4]. Shigella invades the mucosa, causing inflammatory destruction of the large intestinal epithelium, which can result in environmental enteropathy, chronic malnutrition, and cognitive and developmental impairment.

Shigella is known primarily as a cause of bacillary dysentery (blood/mucus in stool, which can be associated with fever and abdominal cramps). However, a large proportion (~40% to 89%) of Shigella-associated diarrhea cases are non-dysentery or secretory diarrhea in nature [5,6,7,8,9]. The clinical syndrome of dysentery caused by Shigella is readily identified clinically and warrants treatment with antibiotics, but Shigella infections causing watery diarrhea are not easily clinically recognized, and the clinical identification of these patients is difficult [7]. The current World Health Organization (WHO) guidelines for the treatment of diarrhea recommend antibiotics when there is the presence of visible blood in diarrhea stool [10,11]. Therefore, cases of non-dysentery Shigella-associated diarrhea (NDSD) are generally not treated with antibiotics.

Oral ciprofloxacin and azithromycin are considered first-line therapeutics for dysentery shigellosis (DS) in adults and children [12]. With effective antibiotic therapy for DS, clinical improvement occurs within 24 to 48 h, resulting in a decreased risk of serious complications and death, shortening the duration of symptoms, and reducing fecal carriage from ~4 weeks to 3 days, while also reducing transmission [12,13,14,15,16]. Thus, the current WHO guidelines appear to manage dysentery effectively but might miss opportunities to reduce mortality and long-term growth potential among children infected with Shigella who present with watery diarrhea.

It may be hypothesized that NDSD cases, if identified quickly, should be treated with antibiotics or other therapeutics to improve the survival and long-term developmental potential of the children. Shigellosis cannot be distinguished reliably from other causes of bloody diarrhea or other causes of watery diarrhea based on clinical features alone. Documenting NDSD infections and rationally guiding treatment will require a point-of-care (POC) test for Shigella so that the treatment can be initiated promptly. Chakraborty et al. previously developed and successfully evaluated, in Asian and African countries, a simple molecular diagnostic test for Shigella: the Rapid LAMP-based Diagnostic Test (RLDT), which detects Shigella from a fecal sample within one hour and thus has the potential to be used for the rapid detection and treatment of NDSD [17,18,19,20].

We are conducting a prospective longitudinal case–control study (INSIGHT) in Mirzapur, Tangail, Bangladesh, to determine if there is a need to change the current guidelines on shigellosis treatment for improving the survival and development of children with this condition. We will determine the impact of NDSD on immediate and long-term (1-year) morbidity and development in children under 5 years old compared with DS and non-Shigella watery diarrhea (WD). We will also assess the potential for implementation of the RLDT for Shigella in a rural primary healthcare government facility in Bangladesh.

## 2. Materials and Methods

### 2.1. Ethical Approval

The protocol for the INSIGHT study was developed through a collaborative effort among the researchers from Johns Hopkins University and the International Centre for Diarrhoeal Disease Research, Bangladesh (icddr,b). The INSIGHT study protocol was reviewed and approved by the Institutional Review Board of the Johns Hopkins Bloomberg School of Public Health and the Ethical Review Committee of the icddr,b. Written informed consent from the parents/caregivers will be obtained by study staff before any study procedures are carried out and confidentiality will be maintained throughout the study period. The results of this study will be submitted for publication in peer-reviewed journals.

INSIGHT is a prospective case–control study including 592 children under 5 years of age who are residents of Tangail seeking care in the Kumudini Hospital (KH) with NDSD, DS, or WD. Children will be given a certain standard of care by the hospital physicians as per the WHO and Bangladesh treatment guidelines. All children will be rehydrated with either intravenous therapy or ORS depending on the degree of their dehydration, and zinc will be provided. Children with DS will be treated with azithromycin or other antibiotics as recommended by the hospital’s attending physician.

### 2.2. Aims

The objectives of this study are to (1) determine the morbidity, risk of hospitalization, and mortality associated with NDSD cases; (2) study the impact of NDSD on the nutritional status and cognitive development of children; (3) understand the impact of NDSD on gut barrier function, systemic inflammation, and gut inflammation in children; (4) determine the Shigella isolates’ resistance to commonly used antibiotics using disk diffusion tests and E-strips; and (4) evaluate if the RLDT could be applicable for the detection and treatment of shigellosis cases in the low-resource clinical settings of a rural primary healthcare facility. The data from aims (1) to (3) will be compared between the NDSD group and the other two groups, DS and WD.

### 2.3. Study Setting and Population

The study children will be enrolled in the KH and followed through twice-weekly home visits. The KH is a nonprofit hospital located in the central urban union of Mirzapur, providing healthcare services to the surrounding poor rural population. Mirzapur is a rural sub-district (Upazila) of Bangladesh that covers 374 square km in the Tangail district. It is located 60 km northwest of the capital city, Dhaka.

### 2.4. Study Design

**Figure 1:**
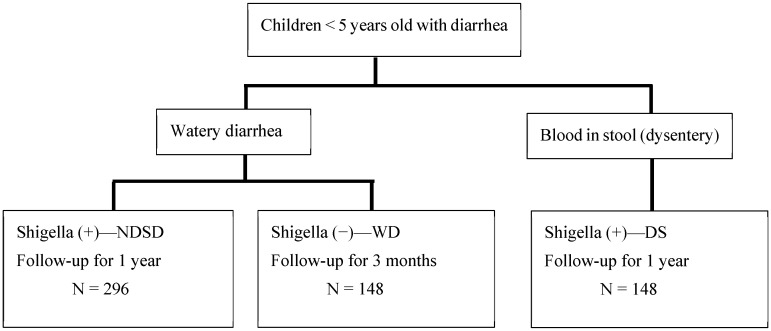
Enrolment flow chart.

Children between 1 and 59 months with acute watery diarrhea, presenting to the KH, with a stool sample that is positive for Shigella according to the RLDT, will be enrolled in the study (named the NDSD group). For comparison, a second group of children with dysentery (presence of visible blood in stool) and positive for Shigella according to the RLDT (DS group) will be included (see enrolment flow chart in Figure 1). The DS group will be treated with antibiotics as a standard treatment. A third group of children with watery diarrhea negative for Shigella according to the RLDT (WD group) will also be enrolled. All RLDT-positive stool samples and 10% of the RLDT-negative stool samples selected at random will be confirmed for Shigella via culture analysis. All RLDT-positive stool samples will be re-tested by qPCR.

Following enrolment, the children in all three groups will be evaluated and compared based on the following factors to assess morbidity: whether requires hospitalization (inpatient), the severity of their diarrhea (the number of loose stools per day, the presence of sunken eyes, the loss of skin turgor, and whether intravenous hydration is required), other clinical symptoms (fever, rectal straining, abdominal pain and cramp, anorexia, convulsion, vomiting, etc.), the length of their illness, and their diarrheal severity score [21]. The duration of the shedding of Shigella bacteria in stool following the index diarrhea episode will be evaluated using stool samples collected on every other day until day 14 and then twice per week tested by the RLDT and culture until two consecutive stool samples are negative for Shigella by the RLDT.

During the follow-up period of 1 year for the NDSD and DS groups and 3 months for the WD group, the field workers will visit the households twice per week to collect morbidity data. Data on any episodes of diarrhea after the index diarrhea episode, including shigellosis (DS and NDSD), hospitalizations, and antibiotic use, will be collected during these visits. In addition to these data on future diarrhea episodes, stool samples will be collected every month and tested for Shigella using the RLDT and culture analysis. Parents or primary caregivers of the enrolled children will undergo standardized interviews to solicit demographic, socioeconomic, sanitation- and hygiene-related, epidemiological, and clinical information. Diarrhea will be defined as three or more loose or liquid stools during a 24 h period, and dysentery as one or more loose stools with visible blood. A diarrheal episode will be defined as “new” if the diarrhea definition is met after at least three or more days free of diarrhea or dysentery. Anthropometry (weight, length, mid-upper arm circumference, and head circumference) will be measured upon enrolment in the KH and every month during home visits by the field workers. The cognitive, language, and motor development of the children will be measured using the Bayley-IV for children 1 to 42 months of age and the Wechsler Preschool and Primary Scale of Intelligence (WPPSI-IV) and Ages and Stages Questionnaire for the older children [22]. The Ages and Stages Questionnaire (ASQ-3) is a developmental screening tool that will be used for measuring the motor development of the older children. Children’s behavior will be measured during the cognitive assessments through five Wolke scales based on their response to the assessor in the first 10 min, measuring their approach, emotional tone, cooperation with the assessor, vocalization, and activity level throughout the test. These ratings have been used previously on Bangladeshi children [23]. Home Observation for Measurement of the Environment (HOME) will be conducted once at the first visit after enrolment. Children’s behavioral assessments will be analyzed using Wolke’s Behavior Rating scale [24,25] by observing their behavior during the Bayley or WPPSI test. This assessment includes responsiveness to the examiner, activity level, emotional tone, cooperation with test procedure, and vocalization on a 9-point scale during the test. It has been proven that this scale is sensitive to intervention effects and can pick up group differences in Bangladeshi cohorts [24]. Two trained female assessors will administer the Bayley-IV, WPPSI, ASQi (motor component), and Behavior Rating tests in the field office through direct assessment, observations, and maternal interviews two days after the index diarrhea episode is resolved and at the 3- and 12-month follow-up visits. The assessors will receive a month-long extensive training on all of the developmental measures by the senior psychologist of the study and will be allowed to carry out data collection after achieving >85% inter-rater agreement with the trainer. To ensure data quality throughout the study period, the senior psychologist will conduct ongoing reliability checks of 10% of the total sample and ensure that refresher training is provided quarterly. Two female enumerators will collect the HOME data at the household level along with other demographic information.

For the detection of Shigella by culture analysis, stool samples will be cultured on MacConkey agar and Salmonella–Shigella agar followed by biochemical tests and then serotyped using commercially available antisera (Denka Seiken, Tokyo, Japan). The Shigella isolates will be tested for their susceptibility to ciprofloxacin, azithromycin, cefixime, ceftriaxone, trimethoprim/sulfamethoxazole, nalidixic acid, ampicillin, and pivmecillinam using the Kirby–Bauer disk diffusion method and the minimum inhibitory concentrations will be determined using E-strips. The isolates will be stored in glycerol stock.

### 2.5. Inclusion and Exclusion Criteria

The inclusion criteria are as follows: (1) children between >1 month and <60 months of age seeking care at the KH for diarrhea; and (2) children residing in the catchment area of the KH and willing to be available for sample and data collection during the scheduled visits. In addition, children in the NDSD and DS groups with stool samples positive for Shigella according to the RLDT will be included. The exclusion criteria are as follows: (1) children whose diarrhea episode started more than 72 h before enrolment; (2) children who have taken antibiotics within the last 3 days; (3) children with severe acute malnutrition (below the −3 Z score of the median WHO growth standards); and (4) children with another significant disease process requiring specific therapy. In addition, for the NDSD and WD groups, children showing the presence of visible blood in their stool will be excluded.

### 2.6. Collection, Preparation, and Archiving of Biological Samples

Stool and blood specimens will be collected at the baseline during enrolment at the hospital by the study staff and during follow-up home visits by the field staff following the schedule (Table 1) to evaluate inflammatory and immune markers. Gut barrier function will be assessed using the lactulose rhamnose (LR) permeability test, wherein urine will be collected before, 2 h after, and 5 h after the administration of LR solution to determine the ratio of L:R among the study children. Using blood serum, we will measure C-reactive protein, intestinal fatty-acid-binding protein, lipopolysaccharide, and flagellar (FliC) IgA and IgG levels to measure systemic inflammation, enterocyte death, and the translocation of pathogens. We will monitor gut inflammation by measuring the levels of myeloperoxidase (MPO) and lactoferrin (LF) in stool samples. The panel of 10 inflammatory cytokines (IL-2, -4, -6, -8, -10, -13, -1ß, TNF-α, IFN-γ, IL-17) will be tested in both serum and stool samples. Other major enteric pathogens will be detected in stool using quantitative PCR. All samples will be collected, processed, and stored following the standard operating procedures developed in the study protocol and following the schedule of events (Table 1).

In order to evaluate whether the RLDT could be implemented in a rural primary healthcare hospital in Bangladesh, we will train the hospital staff in the RLDT procedure and certify them. During the screening for enrolment in the INSIGHT study, 225 randomly selected stool samples will be sent to the rural hospital lab to be rescreened with the RLDT by the trained hospital staff, independently. The results will be compared with the RLDT results that were obtained by the INSIGHT study staff for the purpose of enrolling participants. We will determine the level of technical support needed for the RLDT, its ease of use, and its acceptability among the hospital lab staff using a questionnaire.

## 3. Statistical Considerations

### 3.1. Sample Size and Power

This study’s hypothesis is that the rates of hospitalization (inpatient) will be similar among the NDSD and DS cases. This is a matched set of cases (DS) and controls (NDSD) with two matched controls per case. The second comparison group is the WD group (1:1 matched with case). Based on previous data on the hospitalization rates of shigellosis cases in Mirzapur (obtained via personal communication with Faruque et al.), we assume that the probability of hospitalization among the controls will be 20%. Since it is not known, as a general practice, the correlation coefficient for exposure between the matched cases and controls is set to 0.2. If the true odds ratio for hospitalization in the exposed subjects relative to the unexposed subjects is 0.5, we will need to study 123 cases of shigellosis with dysentery with 2 matched controls per case in order to be able to reject the null hypothesis that this odds ratio equals 1 with a probability (power) of 0.8. If the alternative hypothesis is not satisfied, then we will accept the null hypothesis of no difference in hospitalization rates between the cases and controls. The Type I error probability associated with the test of this null hypothesis is 0.05. With an estimated 20% of participants lost to follow-up, we will enroll 148 children each in the DS and WD groups and 296 in the NDSD group (total: 592).

For the RLDT evaluation in the primary healthcare facility, a sample size of 225 (45 subjects with Shigella) achieves 80% power to detect a change in sensitivity from 0.60 to 0.80 using a two-sided binomial test and 100% power to detect a change in specificity from 0.60 to 0.80 using a two-sided binomial test with the target significance level set to 0.05. The actual significance level achieved by the sensitivity test is 0.0336 and that achieved by the specificity test is 0.0397. The prevalence of the disease is ~20%.

### 3.2. Statistical Analyses

The indicators of severity and symptoms determined by the morbidity indicators, hospitalization, and diarrheal disease score between the cases and controls at baseline (i.e., upon enrolment) will be evaluated using conditional logistic regression adjusting for sex and other confounding factors. The impact of the initial shigellosis or WD episode on the future morbidity, hospitalization, and mortality of the children, during the follow-up period of 12 months for the NDSD and DS groups or 3 months for the WD group, will be analyzed accounting for the confounding factors. Any antibiotic use during the follow-up period will be recorded and considered during analysis. Initially, bivariate conditional logistic regression models will be developed considering each one of the sociodemographic factors for the risk of shigellosis to select the variables for adjustment in the multivariable model.

The Z scores of all anthropometric measurements of the cases and their matched controls at different time intervals will be compared using paired *t*-tests. We will perform linear regression analyses comparing the baseline Z scores and changes in the Z scores from enrolment to the 3- or 12-month follow-up assessment, adjusting for the enrolment Z score, the duration of the follow-up period, sociodemographic covariates, antibiotic use, future diarrhea episodes, and any hospitalization in the follow-up period, using jack-knife estimates of standard error [26].

The same analytic techniques will be used for evaluating the data from the Bayley and WPPSI scales by comparing both the Z scores and age-adjusted composite scores of all developmental domains in the unadjusted model. In the multivariable-adjusted liner regression model, we will adjust specific baseline test scores for each domain and any other related sociodemographic variables that are significantly different between the groups at baseline, significantly different between the children lost to follow up and the tested children, or significantly associated with developmental outcome measures, e.g., socioeconomic status, sex, mother’s education, HOME assessment result, etc. Five behavioral outcomes will be summed up as total scores and will be used in a similar analysis. Sensitivity and mediation analyses will also be conducted using a Structural Equation Model (SEM) [27] to explore the underlying mechanisms or processes influencing the outcomes.

The magnitudes and kinetics of the inflammatory markers will be compared at enrolment and over the follow-up period between DS cases and NDSD cases. Analyses for comparisons of dichotomous outcomes, such as fold increases/decreases in the markers from baseline, will be performed with the chi-square test or Fisher’s exact test if cell counts are sparse. For comparisons of the levels at a specific day between the two groups, a student’s *t*-test will be performed. MPO and LF data will be adjusted for breastfeeding. The data from the cases and their matched controls will be evaluated using paired *t*-tests. Linear regression analysis results will be compared between cases and controls adjusting for anthropometry and cognitive developmental changes, future diarrhea episodes, and antibiotic use in the follow-up period, and sociodemographic risk factors for inflammation.

For the RLDT evaluation at the rural primary healthcare facility, the RLDT results from the hospital staff will be compared with the results from the INSIGHT study staff (gold standard), analyzing sensitivity, specificity, and positive and negative predictive values. We will also use pairwise comparisons with Cohen’s kappa (measuring the agreement between binary outcomes +/− of tests without requiring a gold-standard).

## 4. Discussion

INSIGHT is a case–control, prospective, longitudinal study that aims to understand if the currently recommended therapy of rehydration along with zinc, without antibiotics, is adequate for NDSD cases. In one systemic review, five (71%) of seven studies examining Shigella mortality relative to other causes of diarrhea found the odds of death to be significantly higher in children with Shigella infection than in those without Shigella infection (pooled OR: 2.8, 95% CI: 1.6–4.8; *p* = 0.000); however, dysentery was not associated with mortality (pooled OR: 1.3, 95% CI: 0.7–2.3; *p* = 0.37) [7,28]. Also, a case–control study in severely malnourished children in Bangladesh assessing Shigella mortality stratified by the presence of dysentery found no significant difference between inpatients with DS and those with NDSD in the association between Shigella infection and death. These findings of lower mortality among DS children are likely the consequence of effective management strategies, including the administration of antibiotics for dysentery cases. Although the variability of populations, study designs, diagnostic tools, clinical management strategies, enrolment periods, and sample sizes used in the literature have resulted in marked heterogeneity in the magnitude of this association across studies, overall, systematic reviews have found Shigella infection to be associated with mortality in children presenting with diarrhea and that dysentery (the presence of blood in stool) does not adequately identify children with Shigella infections. The implication is that the exclusion of NDSD from treatment recommendations might leave many children vulnerable to acute and subacute adverse clinical manifestations and developmental delays. The INSIGHT study will determine the impact of NDSD following rehydration therapy (the recommended therapy for watery diarrhea) on morbidity, hospitalization, and gut health in children compared to DS cases who are treated with antibiotics. This study will also evaluate the consequences of NDSD, DS, and WD on children’s physical and mental developmental domains, as there are limited and inconsistent data available in this area [29,30,31,32].

Regarding the management of diarrhea, although the WHO recommends antibiotics only for children with bloody diarrhea, over 40% of children with non-bloody watery diarrhea currently receive antibiotics as part of non-standard treatment in low- and middle-income countries [33]. This overuse of antibiotics contributes to the development and spread of antimicrobial resistance, both at the individual level and at the population level. If the INSIGHT study determines that NDSD cases should be treated with antibiotics to improve the survival and long-term developmental potential of children with this condition, the identification of such cases will require a rapid test to document these infections so that treatment can be evidence-based and initiated promptly.

Shigellosis cannot be distinguished reliably from other causes of bloody diarrhea or other causes of watery diarrhea based on clinical features alone. The most frequently used and the current gold-standard diagnostic method for Shigella is the direct culture method [5,34]. However, recent studies have shown that the culture method is not sufficiently sensitive [8,35]. Moreover, culture analysis takes 2–3 days and is therefore not feasible as a POC diagnostic test. The PCR or quantitative PCR (qPCR) methods are highly sensitive [8,35]; however, these assays are highly dependent on technology and equipment, require improved laboratory facilities and trained personnel, and are time-consuming and expensive. Therefore, the current diagnostic methods in practice are not practical for routine use and are not applicable in most healthcare settings where Shigella infections are endemic. The INSIGHT study will evaluate the performance and implementation of the simple, rapid, and sensitive RLDT assay for Shigella in a primary healthcare facility in Bangladesh in order to determine if the RLDT could be used as a potential diagnostic tool to rapidly detect and treat shigellosis. Identifying children with shigellosis and developing targeted treatment strategies for these children may potentially lower antimicrobial resistance.

## Figures and Tables

**Table 1:** Schedule of events.

Study Day/Month (M)	1	3	5	9	14	M1	M2	M3	M4	M5	M6	M7	M8	M9	M10	M11	M12
Enrolment	X																
* Morbidity	X	X	X	X	X	X	X	X	X	X	X	X	X	X	X	X	X
* Stool collection for analysis of microbiology	X	X	X	X	X	X	X	X	X	X	X	X	X	X	X	X	X
Sociodemographic, sanitation, and hygiene questionnaire assessments		X									X						
Urine L:R ratio measurement		X			X			X			X						X
Stool collection for analysis of inflammatory/immune markers	X	X	X	X	X	X		X			X			X			X
Blood collection for analysis of inflammatory/immune markers	X			X		X		X			X			X			X
Anthropometry measurements	X				X	X	X	X	X	X	X	X	X	X	X	X	X
Bayley or WPPSI assessment		X						X									X
HOME assessment		X															

“X” marked the time points of collecting data and samples. * Morbidity data and stool samples will be collected every other day following the baseline visit until day 14 and then two days per week until the participant with DS or NDSD is negative for Shigella. Once their stool has tested negative for Shigella, morbidity data will be collected during home visits twice a week and stool samples will be collected as per the study schedule.

## Data Availability

The datasets generated during the current study are available from the corresponding author upon reasonable request.

## References

[1] Khalil I.A., Troeger C., Blacker B.F., Rao P.C., Brown A., Atherly D.E., Brewer T.G., Engmann C.M., Houpt E.R., Kang G. (2018). Morbidity and mortality due to shigella and enter-otoxigenic *Escherichia coli* diarrhoea: The Global Burden of Disease Study 1990–2016. Lancet Infect Dis..

[2] Anderson J.D., Bagamian K.H., Muhib F., Amaya M.P., Laytner L.A., Wierzba T., Rheingans R. (2019). Burden of enterotoxigenic *Escherichia coli* and Shigella non-fatal diarrhoeal infections in 79 low-income and lower middle-income countries: A modelling analysis. Lancet Glob. Health.

[3] Murray C.J., Barber R.M., Foreman K.J., Abbasoglu O.A., Abd-Allah F., Abera S.F., Aboyans V., Abraham J.P., Abubakar I., GBD 2013 DALYs and HALE Collaborators (2015). Global, regional, and national disability-adjusted life years (DALYs) for 306 diseases and injuries and healthy life expectancy (HALE) for 188 countries, 1990–2013: Quantifying the epidemiological transition. Lancet.

[4] Murray C.J.L., Vos T., Lozano R., Naghavi M., Flaxman A.D., Michaud C., Ezzati M., Shibuya K., Salomon J.A., Abdalla S. (2012). Disability-adjusted life years (DALYs) for 291 diseases and injuries in 21 regions, 1990–2010: A systematic analysis for the Global Burden of Disease Study 2010. Lancet.

[5] Kotloff K.L., Nataro J.P., Blackwelder W.C., Nasrin D., Farag T.H., Panchalingam S., Wu Y., Sow S.O., Sur D., Breiman R.F. (2013). Burden and aetiology of diarrhoeal disease in infants and young children in developing countries (the Global Enteric Multicenter Study, GEMS): A prospective, case-control study. Lancet.

[6] Kotloff K.L., Nasrin D., Blackwelder W.C., Wu Y., Farag T., Panchalingham S., Sow S.O., Sur D., Zaidi A.K., Faruque A.S. (2019). The incidence, aetiology, and adverse clinical consequences of less severe diarrhoeal episodes among infants and children residing in low-income and mid-dle-income countries: A 12-month case-control study as a follow-on to the Global Enteric Multicenter Study (GEMS). Lancet Glob. Health.

[7] Tickell K.D., Brander R.L., Atlas H.E., Pernica J.M., Walson J.L., Pavlinac P.B. (2017). Identification and management of Shigella infection in children with diarrhoea: A systematic review and meta-analysis. Lancet Glob. Health.

[8] Liu J., Platts-Mills J.A., Juma J., Kabir F., Nkeze J., Okoi C., Operario D.J., Uddin J., Ahmed S., Alonso P.L. (2016). Use of quantitative molecular diagnostic methods to identify causes of diarrhoea in children: A reanalysis of the GEMS case-control study. Lancet.

[9] Lindsay B., Saha D., Sanogo D., Das S.K., Omore R., Farag T.H., Nasrin D., Li S., Panchalingam S., Levine M.M. (2015). Association Between Shigella Infection and Diarrhea Varies Based on Location and Age of Children. Am. J. Trop. Med. Hyg..

[10] World Health Organization (2005). Guidelines for the Control of Shigellosis, Including Epidemics Due to Shigella Dysenteriae Type 1. https://www.who.int/publications/i/item/9241592330.

[11] World Health Organization (2013). Pocket Book of Hospital Care for Children.

[12] Kotloff K.L., Riddle M.S., Platts-Mills J.A., Pavlinac P., Zaidi A.K.M. (2018). Shigellosis. Lancet.

[13] Gu B., Cao Y., Pan S., Zhuang L., Yu R., Peng Z., Qian H., Wei Y., Zhao L., Liu G. (2012). Comparison of the prevalence and changing resistance to nalidixic acid and ciprofloxacin of Shigella between Europe–America and Asia–Africa from 1998 to 2009. Int. J. Antimicrob. Agents.

[14] Williams P.C.M., Berkley J.A. (2018). Guidelines for the treatment of dysentery (shigellosis): A systematic review of the evidence. Paediatr. Int. Child Health.

[15] Das J.K., Ali A., Salam R.A., Bhutta Z.A. (2013). Antibiotics for the treatment of cholera, shigella and cryptosporidiumin children. BMC Public Health.

[16] Christopher P.R., David K.V., John S.M., Sankarapandian V. (2010). Antibiotic therapy for shigella dysentery. Cochrane Database Syst. Rev..

[17] Chakraborty S., Connor S., Velagic M. (2022). Development of a simple, rapid, and sensitive diagnostic assay for enterotoxigenic *E. coli* and *Shigella* spp applicable to endemic countries. PLoS Neglected Trop. Dis..

[18] Silwamba S., Chilyabanyama O.N., Liswaniso F., Chisenga C.C., Chilengi R., Dougan G., Kwenda G., Chakraborty S., Simuyandi M. (2022). Field evaluation of a novel, rapid diagnostic assay, and molecular epidemiology of enterotoxigenic *E. coli* among Zambian children presenting with diarrhea. PLoS Neglected Trop. Dis..

[19] Chowdhury G., Ghosh D., Zhou Y., Deb A.K., Mukhopadhyay A.K., Dutta S., Chakraborty S. (2024). Field evaluation of a simple and rapid diagnostic test, RLDT to detect Shigella and enterotoxigenic *E. coli* in Indian children. Sci. Rep..

[20] Héma A., Sermé S.S., Sawadogo J., Diarra A., Barry A., Ouédraogo A.Z., Nébié I., Tiono A.B., Houard S., Chakraborty S. (2023). Contribution of the Rapid LAMP based Diagnostic Test (RLDT) to the evaluation of enterotoxi-genic *Escherichia coli* (ETEC) and Shigella in childhood diarrhea in the peri-urban area of Ouagadougou, Burkina Faso. Microorganisms.

[21] Lee G.O., Richard S.A., Kang G., Houpt E.R., Seidman J.C., Pendergast L.L., Bhutta Z.A., Ahmed T., Mduma E.R., Lima A.A. (2016). A Comparison of Diarrheal Severity Scores in the MAL-ED Multisite Community-Based Cohort Study. J. Pediatr. Gastroenterol. Nutr..

[22] Tofail F., Hamadani J.D., Mehrin F., Ridout D.A., Huda S.N., Grantham-McGregor S.M. (2013). Psychosocial stimulation benefits de-velopment in nonanemic children but not inanemic, iron-deficient children. J. Nutr..

[23] Hamadani J.D., Huda S.N., Khatun F., Grantham-McGregor S.M. (2006). Psychosocial stimulation improves the development of undernourished children in rural bangladesh. J. Nutr..

[24] Hamadani J.D., Mehrin S.F., Tofail F., Hasan M.I., Huda S.N., Baker-Henningham H., Ridout D., Grantham-McGregor S. (2019). Integrating an early childhood development programme into Bangladeshi primary health-care services: An open-label, cluster-randomised controlled trial. Lancet Glob. Health.

[25] Wolke D., Skuse D., Mathisen B. (1990). Behavioral style in failure-to-thrive infants: A preliminary communication. J. Pediatr. Psychol..

[26] Efron B. (1981). Nonparametric Estimates of Standard Error: The Jackknife, the Bootstrap and Other Methods. Biometrika.

[27] Jensen S.K.G., Kumar S., Xie W., Tofail F., Haque R., Petri W.A., Nelson C.A. (2019). Neural correlates of early adversity among Bangladeshi infants. Sci. Rep..

[28] van den Broek J.M., Roy S.K., Khan W.A. (2005). Risk factors for mortality due to shigellosis: A case-control study among severe-ly-malnourished children in Bangladesh. J. Health Popul. Nutr..

[29] MacIntyre J., McTaggart J., Guerrant R.L., Goldfarb D.M. (2014). Early childhood diarrhoeal diseases and cognition: Are we missing the rest of the iceberg?. Paediatr. Int. Child Health.

[30] Piper J.D., Chandna J., Allen E., Linkman K., Cumming O., Prendergast A.J., Gladstone M.J. (2017). Water, sanitation and hygiene (WASH) in-terventions: Effects on child development in low- andmiddle-income countries. Cochrane Database Syst. Rev..

[31] Pinkerton R., Patrick P.D., Moore S.R., Wiseman B.L., Niehaus M.D., Oriá R.B., Guerrant R.L., Lima A.A.M., Rogawski E.T., Oriá M.O.B. (2016). Early Childhood Diarrhea Predicts Cognitive Delays in Later Childhood Independently of Malnutrition. Am. J. Trop. Med. Hyg..

[32] Lorntz B., Soares A.M., Moore S.R., Pinkerton R., Gansneder B., Bovbjerg V.E., Guyatt H., Lima A.M., Guerrant R.L. (2006). Early childhood diarrhea predicts impaired school performance. Pediatr. Infect. Dis. J..

[33] Rogawski E.T., Platts-Mills J.A., Seidman J.C., John S., Mahfuz M., Ulak M., Shrestha S.K., Soofi S.B., Yori P.P., Mduma E. (2016). Use of antibiotics in children younger than two years in eight countries: A prospective cohort study. Bull. World Health Organ..

[34] Platts-Mills J.A., Babji S., Bodhidatta L., Gratz J., Haque R., Havt A., McCormick B.J., McGrath M., Olortegui M.P., Samie A. (2015). Pathogen-specific burdens of community diarrhoea in developing countries: A multisite birth cohort study (MAL-ED). Lancet Glob. Health.

[35] Lindsay B., Pop M., Antonio M., Walker A.W., Mai V., Ahmed D., Oundo J., Tamboura B., Panchalingam S., Levine M.M. (2013). Survey of culture, goldengate assay, universal biosensor assay, and 16S rRNA gene sequencing as alternative methods of bacterial pathogen detection. J. Clin. Microbiol..

